# Supplementary material for: Hit Compounds and Associated Targets in Intracellular Mycobacterium tuberculosis
Source: Molecules. 2022 Jul 12;27(14):4446. doi: 10.3390/molecules27144446 (PMC9324642; doi:10.3390/molecules27144446)
Supplement: Supplementary file 1 [file molecules-27-04446-s001.zip › molecules-1795141-supplementary.pdf]

Table S1. Summary of statistics on genomes of chemical mutants.

Compound name	Isolate no.	No. of reads	Average read length after quality trimming	Average Coverage (×)	SRA number
**213A**	M213A-1	641063	221.361	60	SAMN12492977
**213A**	M213A-3	1051601	204.572	91	SAMN12492978
**213A**	M213A-4	727432	209.653	65	SAMN12492979
**213A**	M213A-5	832402	204.491	75	SAMN12492980
**213A**	M213A-6	1803775	201.447	159	SAMN12492981
**267A**	M267A-1	801260	205.132	71	SAMN12492989
**267A**	M267A-2	1261987	213.699	118	SAMN12492990
**267A**	M267A-3	712871	239.031	75	SAMN12492991
**290A**	M290A-1	994162	207.967	89	SAMN12493002
**290A**	M290A-2	679313	212.892	62	SAMN12493003
**950A**	M950A-1	947041	192.241	77	SAMN12492982
**950A**	M950A-2	734506	209.301	67	SAMN12492983
**950A**	M950A-3	854282	210.533	77	SAMN12492984
**950A**	M950A-4	2071066	206.255	186	SAMN12492985
**739A**	M739A-1	663236	213.818	60	SAMN12492986
**739A**	M739A-2	1003468	211.514	92	SAMN12492987
**739A**	M739A-5	712569	215.105	67	SAMN12492988
**472A**	M472A-1	596851	214.151	55	SAMN12492992
**472A**	M472A-2	628757	215.148	59	SAMN12492993
**472A**	M472A-3	850420	204.471	76	SAMN12492994
**472A**	M472A-4	867434	206.796	77	SAMN12492995
**412A**	M412A-1	777226	208.215	70	SAMN12493021
**412A**	M412A-2	855342	200.229	74	SAMN12493022
**412A**	M412A-g2	617853	211.919	55	SAMN12493023
**412A**	M412A-g3	331156	227.985	32	SAMN12493024
**412A**	M412A-g4	397877	210.416	36	SAMN12493025
**412A**	M412A-g5	478677	207.938	43	SAMN12493026
**412A**	M412A-g6**	151782	217.829	14	SAMN12493027
**412A**	M412A-g1	655274	226.13	65	SAMN12493028
**412A**	M412A-96w	615279	213.647	57	SAMN12493029

**296A**	M296A-1	750658	229.168	75	SAMN12492996
**296A**	M296A-2	375308	221.593	36	SAMN12492997
**296A**	M296A-3	436737	225.236	43	SAMN12492998
**296A**	M296A-5	307959	224.015	30	SAMN12492999
**296A**	M296A-6	391815	216.327	36	SAMN12493000
**296A**	M296A-8	383941	215.392	35	SAMN12493001
**648X**	M648X-1	439381	225.1	43	SAMN12493004
**648X**	M648X-2	263481	196.2	23	SAMN12493005
**454A**	M454A-3	694228	213.408	64	SAMN12493008
**454A**	M454A-1c	484348	213.19	45	SAMN12493006
**454A**	M454A-2c	280586	209.326	25	SAMN12493007
**1114A**	M1114A-3	635142	229.79	64	SAMN12493010
	M1114A-4	641429	240.215	67	SAMN12493011
**486X**	M486X-1	727728	224.1	71	SAMN12493012
	M486X-2	669706	228.923	67	SAMN12493013
	M486X-3	501664	215.511	47	SAMN12493014
**912A**	M912A-1	617723	235.744	64	SAMN12493015
	M912A-2	665069	232.186	67	SAMN12493016
	M912A-3	697851	240.988	73	SAMN12493017
**622A**	M622A-1	694522	233.749	71	SAMN12493018
	M622A-2	528977	228.252	53	SAMN12493019
	M622A-3	691163	234.247	71	SAMN12493020
**705A**	M705A-5	1026520	244.6	101	SAMN13675147

Table S2. A list of variants in all mutants. High quality SNPs were visually examined and for those with quality score satisfied the criteria: (i) homozygous variants – the alternative allele in strains/mutants are different from the reference genome H37Rv (lab WT); (ii) the coverage of the variant is $\geq$ 30; (iii) the variant receives high confidence with low error rate

Compound	Mutant#	Average Depth	Gene	Genome position	Gene Position | Phred Quality Score | Effect
213A	1	60	*mmpL3*	246564	755A>G | 3324 | Tyr252Cys codon change
213A	3	91	*mmpL3*	246561	758G>A | 4181 | Gly253Glu codon change
213A	4	65	*mmpL3*	246444	875T>C | 3033| Ile292Thr codon change

213A	5	74	*mmpL3*	245268	2051T>C |3167 | Val684Ala codon change
213A	6	159	*mmpL3*	245334	1985C>A | 6990 | Ala662Glu codon change
267A	1	71	*mmpL3*	246554	765C>G | 3099 | Phe255Leu codon change
267A	2	118	*mmpL3*	246556	763T>C |5677 | Phe255Leu codon change
267A	3	75	*mmpL3*	245387	1932 C>A |2510| Phe644Leu codon change
290A	1	88.6	*Rv0370c*	447570	474G>T | 4216 | Val158Val silent
			mmpL3	245410	1909C>A | 3945 | Leu637Ile codon change
290A	2	61.9	*Rv0370c*	447570	474G>T | 3480 | Val158Val silent
			mmpL3	245410	1909C>A | 2969 | Leu637Ile codon change
950A	1	77.3	*Rv3629c*	4068414	641G>A | 3645 | Gly214Glu codon change
			rpoB	761526	1720G>A | 3109 | Asp574Asn codon change
950A	2	66.5	*Rv3629c*	4068414	641G>A | 3162 | Gly214Glu codon change
			rpoB	761526	1720G>A | 2350 | Asp574Asn codon change
950A	3	76.6	*Rv3629c*	4068414	641G>A | 3233 | Gly214Glu codon change
			rpoB	761526	1720G>A | 2847 | Asp574Asn codon change
950A	4	185	*Rv3629c*	4068414	641G>A | 7143 | Gly214Glu codon change
			rpoB	761526	1720G>A | 7165 | Asp574Asn codon change
739A	1	60.4	*ethA*	4326863	611T>C | 1473 | Met204Thr codon change
			Rv3220c	3596788	746C>CA | 2075.97 | indels, frameshift_variant
739A	2	92.4	*ethA*	4326863	611T>C | 2375 | Met204Thr codon change
			Rv3220c	3596788	746C>CA | 4643.97 | indels, frameshift_variant
739A	5	66.7	*rpsO*	3093592	157C>T | 2910 | Arg53Trp codon change
			Rv1024	1146011	154C>T | 1488 | Pro52Ser codon change
472A	1	55.4	*ethA*	4326863	611T>C | 1804 | Met204Thr codon change

			Rv3220c	3596788	746C>CA | 3463.97 | indels, frameshift_variant
472A	2	58.6	*ethA*	4326863	611T>C | 2242 | Met204Thr codon change
			Rv3220c	3596788	746C>CA | 1906.97 | indels, frameshift_variant
472A	3	75	*ethA*	4326863	611T>C | 2422 | Met204Thr codon change
472A	4	76.8	*rpsO*	3093592	157C>T | 3658 | Arg53Trp codon change
			Rv3220c	3596788	746C>CA | 3758.97 | indels, frameshift_variant
412A	1	70	*prrB*	1005390	452T>C | 2719 | Leu151Pro codon change
			moaC3	3709857	392A>G | 2636 | Asp131Gly codon change
412A	2	73.5	*prrB*	1005390	452T>C | 2812 | Leu151Pro codon change
412A	g2	55	*prrB*	1005294	548C>T | 1604 | Thr183Ile codon change
412A	g3	32	*prrB*	1005390	452T>C | 1722| Leu151Pro codon change
412A	g4	35	*prrB*	1005390	452T>C | 1209 | Leu151Pro codon change
			ctpI	127424	3113TGCGAG>T | 2453 | indel, frameshift_variation
412A	g5	42	*prrB*	1005390	452T>C | 1230 | Leu151Pro codon change
			iniB	409651	290C>T | 1237 | Thr97Ile codon change
			narL	940809	298G>C | 2346 | Ala100Pro codon change
412A	g6**	14	*prrB*	1004967	875A>G | 459 | Gln292Arg codon change
412A	g1	65	*prrB*	1005390	452T>C | 2077 | Leu151Pro codon change
412A	96w	57	*prrB*	1005390	452T>C | 1115 | Leu151Pro codon change
296A	1	75	*TB18.5*	193902	277A>G | 2847 | Thr93Ala codon change
296A	2	36	*TB18.5*	193902	277A>G | 1407 | Thr93Ala codon change
			Rv1948c	2198943	122C>A | 1337 | Ala41Glu codon change
296A	3	42	*TB18.5*	193770	145G>C | 1506 | Val49Leu codon change
			pks6	486397	667G>A | 1546 | Val223Ile codon change
296A	5	29	*TB18.5*	193770	145G>C| 1465 | Val49Leu codon change
			pks6	486397	667G>A | 876 | Val223Ile codon change
296A	6	36	*TB18.5*	193861	236A>G | 1630 | Tyr79Cys codon change
296A	8	34	*TB18.5*	193868	243C>A | 1148 | His81Gln codon change
648X	1	42.7			
648X	2	23.1	*ctpC*	3652036	1511C>T | 488 | Ser504Phe codon change

454A	3	64.3	*Rv0678*	779454	466G>GC	1623.97	indels, frameshift_variation
			mbtA	2677303	1369CT>C	1502.97	indels, frameshift_variation
454A	1c	45	*Rv0678*	779454	466G>GC	143.97	indels, frameshift_variation
			mbtA	2677303	1369CT>C	363.97	indels, frameshift_variation
454A	2c	30	*Rv0678*	779454	466G>GC	283.97	indels, frameshift_variation
			mbtA	2677303	1369CT>C	955.97	indels, frameshift_variation
1114A	3	63	*Rv0585c*	683071	1202A>C	2693	Asp401Ala, codon change
		54	*sugI*	3717105	16C>T	1373	Gln6*, stop codon gained
1114A	4	67	*dnaE1*	1749908	2215A>G	1695	Met739Val, codon change
			virS	3447444	983C>A	2484	Pro328His, codon change
		104	*sugI*	3717105	16C>T	3988	Gln6*, stop codon gained
486X	1	71	*phoR*	853056	661G>C	1687	Ala221Pro, codon change
			fbiC	1304012	1082C>A	1868	Thr361Lys, codon change
486X	2	67	*phoR*	853056	661G>C	1559	Ala221Pro, codon change
			fbiA	3641408	866T>A	645	Leu289Gln, codon change
486X	3	47	*phoR*	853056	661G>C	816	Ala221Pro, codon change
			fbiA	3641408	866T>A	1649	Leu289Gln, codon change
			Rv3327	3712046	296C>G	650	Pro100Ala, codon change
912A	1	64	*Rv3083*	3449286	783G>A	1782	Trp261*, gained stop codon
			ethA	4327269	205T>C	2581	Trp69Arg, codon change
912A	2	67	*Rv2542*	2866171	1042G>A	2301	Ala348Thr, codon change
			Rv3083	3449309	806T>C	1392	Leu269Pro, codon change
			ethA	4327284	190T>C	2919	Phe64Ile, codon change
912A	3	73	*Rv2542*	2866171	1042G>A	2275	Ala348Thr, codon change
622A	1	96	*ftsK*	3061315	1192T>C	1462	Ser398Pro, codon change
			virS	3447715	712G>T	1859	Val238Phe, codon change

			Rv3175	3543120	261G>A | 3801 | Lys87Lys, silent
		81	*sugI*	3717105	16C>T | 2583 | Gln6*, stop codon gained
622A	2	83	*sugI*	3717105	16C>T | 2901 | Gln6*, stop codon gained
622A	3	117	*sugI*	3717105	16C>T | 4162 | Gln6*, stop codon gained
705A	1	99	*Rv3083*	3448882	380_381 G>GA|3556| Glu127_Thr128fs, Frame shift variant, indels
		105	*sugI*	3717105	16C>T | 3391 | Gln6*, stop codon gained
